# Electro‐Stimulated Graphene‐Polymer Nanocomposites Enable Wearable Patches With Feedback‐Controlled Drug Release

**DOI:** 10.1002/adhm.202505894

**Published:** 2025-12-29

**Authors:** Santosh K. Misra, Ketan Dighe, Pranay Saha, Teresa Aditya, Muhammad S. Khan, Maha Alafeef, Parikshit Moitra, Dipanjan Pan

**Affiliations:** ^1^ Bioengineering Department University of Illinois at Urbana‐Champaign Urbana USA; ^2^ Department of Biological Sciences & Bioengineering Indian Institute of Technology Kanpur UP India; ^3^ Department of Biomedical Engineering The Pennsylvania State University University Park PA USA; ^4^ Department of Nuclear Engineering The Pennsylvania State University Hallowell Building University Park PA USA; ^5^ Cardiovascular Research and Training Institute The University of Utah Salt Lake City UT USA; ^6^ Biomedical Engineering Department Jordan University of Science and Technology Irbid Jordan; ^7^ Department of Materials Science and Engineering The Pennsylvania State University University Park PA USA; ^8^ Department of Chemistry The Pennsylvania State University University Park PA USA; ^9^ Huck Institutes of Life Sciences Millennium Science Complex The Pennsylvania State University University Park PA USA

**Keywords:** controlled release, graphene, nanomaterial, polymer, real time monitoring, wearable

## Abstract

Stimuli‐responsive nanomaterials capable of spatiotemporal control over drug release are of nanocomposite patch (“e‐Medi‐Patch”) engineered from biodegradable polycaprolactone (PCL), graphene nanoplatelets, and a redox‐active therapeutic, niclosamide. The hierarchical composite integrates π‐π interactions between aromatic drug molecules and conductive graphene to enhance loading and retention, an Au microelectrode interface to enable wireless electrostimulation, and bluetooth‐assisted impedance sensing for real‐time monitoring of release dynamics. Under mild electrical stimulation, the nanocomposite exhibits on‐demand, unidirectional release of niclosamide with tunable kinetics, confirmed by modelling, in vitro melanoma cell studies, and in vivo xenograft tumor regression. Unlike conventional slow‐release patches that rely on passive diffusion, the e‐Medi‐Patch uniquely offers on‐demand electrostimulatory release with real‐time feedback monitoring, transforming drug delivery from a static system into an actively controlled, intelligent therapeutic platform. Beyond melanoma, the platform accommodates other redox‐active therapeutics and offers scalable melt‐blending fabrication. This work establishes an integrated materials‐electronics strategy for wearable, feedback‐controlled drug delivery, bridging multifunctional nanocomposites and precision medicine.

## Graphical abstract

xx xx.

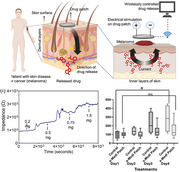

## 1 Introduction

Stimuli‐responsive nanomaterials that couple structural hierarchy with external controllability are central to the development of next‐generation wearable devices and precision therapeutics. Among available stimuli, electrical cues are particularly attractive because they are non‐invasive, tunable, and readily integrable with portable electronics.[1, 2, 3, 4, 5, 6, 7, 8, 9] However, most current electro‐responsive systems rely on conductive polymers or hydrogels, which often suffer from limited stability, uncontrolled passive diffusion, and a lack of real‐time feedback mechanisms. These challenges have hindered the translation of electro‐stimulatory drug release platforms into clinically relevant, user‐friendly technologies.

2D nanomaterials provide unique opportunities for engineering multifunctional composites with tunable electro‐responsiveness. Graphene nanoplatelets combine high surface area, electrical conductivity, and π‐π interactions that enable efficient retention and release of aromatic or redox‐active drugs.[2, 10, 11, 12] When integrated within biodegradable polymer matrices, such as polycaprolactone (PCL), graphene nanoplatelets can establish percolative conductive pathways and modulate drug‐matrix interactions, yielding platforms that are simultaneously biocompatible, structurally robust, and responsive to external fields. Yet, despite progress in graphene‐polymer systems, achieving spatiotemporal control of release kinetics together with quantitative, real‐time monitoring of drug delivery events remains an unmet challenge.

Here, we report a wireless electro‐responsive nanocomposite patch (“e‐Medi‐Patch”) that integrates multiple key functionalities within a scalable design. Hierarchical incorporation of graphene nanoplatelets into a PCL matrix for enhanced drug loading and electrically triggered release can be achieved. Gold microelectrode interfaces enabling low‐voltage electrostimulation, and Bluetooth‐assisted impedance monitoring can be incorporated providing real‐time feedback on drug release kinetics. Using niclosamide, a model redox‐active therapeutic, as a test case, we demonstrate tunable release under mild electrical cues, unidirectional transport, and quantitative monitoring with on‐demand feedback control. The multifunctional design is validated through computational modeling, in vitro cancer cell assays, and in vivo xenograft studies, establishing a broad platform for wearable, feedback‐controlled therapeutics.

While numerous patches have been reported for passive or slow‐release therapy, these systems generally suffer from fixed release rates, lack of external controllability, and an absence of quantitative monitoring.[13, 14] Once applied, such patches cannot dynamically adjust dosage, leading to risks of under‐dosing, overdose, or unintended “dose dumping.” Recent advances include microneedle‐COF patches for melanoma, nanocellulose‐magnetic nanoparticle patches, Au‐deposited polycaprolactone patches for neurological therapy, and two‐electrode microneedle systems for atopic dermatitis.[15, 16, 17] Piezoelectric patches have improved dosage control via mechanical movement, yet achieving controlled, facile, and real‐time release remains a major challenge.[18, 19] In contrast, the e‐Medi‐Patch integrates core advantages like externally triggered and on‐demand release via mild electrical stimulation, enabling precise spatiotemporal control. Real‐time feedback monitoring can be achieved through Bluetooth‐assisted impedance sensing, providing quantitative information on release kinetics. A graphene‐polymer nanocomposite architecture can couple high drug loading with electro‐responsive pathways. These features advance the e‐Medi‐Patch beyond traditional slow‐release platforms toward an intelligent, feedback‐controlled drug delivery platform.

Human skin offers a promising transdermal route for drug delivery that bypasses hepatic metabolism and enables localized therapy. However, while smart biomaterials responsive to heat, pH, light, enzymes, or magnetic fields have advanced this approach, their implementation remains challenging due to the need for complex, specialized equipment.[20, 21, 22] Some conventional transdermal drug delivery approaches include ointments, gels and elastomers which presents major drawbacks of requiring frequent application, skin reactions, inaccuracy of the dosage administered, instability, skin allergies, dryness and lesions.[23, 24, 25, 26, 27] Apart from the inconsistency in drug release, problems with drug loading is also commonly seen in elastomers.[28, 29] Drug loading can often effect the curing or crosslinking properties of elastomers and leaching of unreacted curing agents making them unreliable material in terms of mechanical integrity and toxicity. Prior reports of reservoir‐type or matrix‐type transdermal patches are often made of biologically detrimental materials, e.g. narcotic (Fentanyl) or methyl salicylate, which can cause toxicity issues.[29, 30, 31] The reservoir‐type patches retain drug in a solution or gel form, maintaining rate‐controlling membrane between the drug reservoir and the skin.[32] Matrix‐type patches incorporate drug in an adhesive which can be used directly,[33, 34, 35]assisted by skin permeability alone.[36, 37] Drug‐loaded patches, backing films, microneedles, thermal, mechanical and electrical ablation, have also been proposed without much efficiency.[30, 38, 39, 40, 41]

Graphene is known to be an exceptional semi‐metal conductor where facile electron exchange occurs at the edges or at the defects in the basal plane, [34] which assists in oxidation/reduction processes as well. It also has a relatively large surface area (2630 m^2^/g) among existing nanomaterials.[42, 43, 44] The non‐covalent surface decoration in GO provides targeted delivery, while the delocalized π‐electrons throughout the nanosheet surface enables π‐π interactions with aromatic drug compounds.[34, 45, 46] Despite achieving precise control to target specific locations, such approaches depend on desorption of the drug molecules from the GO nanosheets by either passive or pH/redox‐controlled mechanisms, which limits the ability to monitor and control the drug dosage in real time.[33, 34] Though GO and polymer‐based hydrogels have attempted to control dosage via modulating release rate with external electric field, they involve voltages that may damage biological tissues while drug being passively diffused through their porous morphology. Thus, using graphene‐based nanoplatelets for drug loading and controlled release with electrostimulation could be a feasible strategy only at a higher potential for transdermal therapy.[32, 33, 34, 46, 47] Bioelectronic patches are limited by conventional polymers that block airflow and degrade unpredictably. Flexible systems therefore require conductive, breathable, and biodegradable materials.[48] Polycaprolactone (PCL) meets these demands with tunable degradation, mechanical strength, and high permeability, and its compatibility with electroactive graphene and redox active drugs makes it ideal for next‐generation controllable patches.[49, 50]

This study introduces a biodegradable polycaprolactone (PCL)‐graphene (GR) nanocomposite patch for near‐closed‐loop, real‐time monitoring and controlled delivery of the redox‐active anticancer drug niclosamide (NIC) to cancer stem cell (CSC) targets. NIC, an aromatic STAT3 (Signal Transducer and Activator of Transcription 3) inhibitor, selectively suppresses CSCs through redox modulation.

While a few studies have explored real‐time cancer therapeutic monitoring such as in vivo pH sensors and wearable methotrexate‐delivery patches, these systems remain limited in scope and control.[51, 52] To overcome this, we have developed the electro‐stimulated e‐Medi‐Patch (NIC‐GR‐PCL), a conductive graphene‐PCL scaffold that permits precise, on‐demand NIC release through electrical modulation. Graphene nanoplatelets enhance both drug loading capacity and electrical conductivity, enabling redox‐triggered, electro‐responsive release.[53] The biocompatible and bioresorbable PCL matrix, widely used in biomedical grafts, provides tunable degradation, mechanical robustness, and high permeability, supporting its role as a flexible platform for controlled electroactive delivery.[54]

The resulting on‐demand electrochemical drug delivery system overcomes traditional limitations in drug loading and release control, allowing personalized, tunable dosage through an iontophoretic mechanism.[55] A mild electrical current applied to the skin drives charged drug migration across the epidermal barrier, while electro‐osmotic flow facilitates co‐transport of neutral and weakly charged species.[56] The release efficiency can be precisely modulated by pulse parameters (waveform, duration, amplitude) and by intrinsic drug properties, including partition coefficient (log P), pKa, and solubility.[57]

**FIGURE 1:**
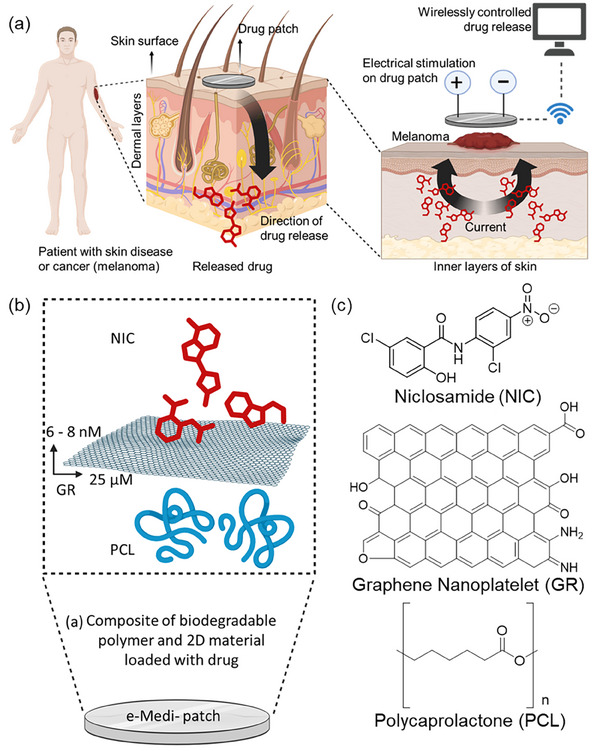
(a) Schematic presenting the plan and feasibility of using an electro‐stimulatory, drug eluting, wirelessly controllable patch for transdermal therapy of patients with cancer and other skin diseases. (b) Schematic description of compositional details of e‐Medi‐Patch. (c) molecular details of the materials used in preparation of e‐Medi‐Patch.

The system regulates both the dosage and duration of release via remotely applied electrical stimulation using a Bluetooth‐enabled controller (Figure 1a). Application of an electric potential effectively triggered on‐demand release of the redox‐sensitive nicotinamide (NIC) drug from the e‐Medi‐Patch, achieving significant therapeutic efficacy against C32 human melanoma cells, without passive diffusion. Electrical pulses transiently reduce skin barrier resistance, enhancing transdermal transport. Integration with gold (Au) microelectrodes enables real‐time impedance and potential monitoring, providing direct electrochemical feedback on drug release.[58] Coupled with a handheld Bluetooth impedance analyzer, the patch achieves wireless, adaptive control of delivery profiles. This multifunctional closed‐loop platform unites controlled electro‐responsive release with quantitative feedback, representing a next‐generation, intelligent, and patient‐tailored drug delivery system.

## 2 Results and discussion

### 2.1 Fabrication and Characterization of Electro‐Responsive Medicinal Patch (e‐Medi‐Patch)

A novel polymer‐graphene composite‐based, drug‐loaded patch was developed to achieve precise control over the delivery of a model drug, niclosamide (NIC), for potential use against melanoma. This e‐Medi‐Patch (NIC‐GR‐PCL) was integrated with an electronic circuit connected to a data analyzer via Bluetooth, enabling real‐time monitoring and analysis of drug release dynamics.

The patch was fabricated using a melt‐blending method (Figure 1b) with polycaprolactone (PCL), graphene nanoplatelets, and NIC at an optimized weight ratio of 100:2:1, respectively (Figure 1c). This composition ensured uniform drug distribution within the composite and optimal printability of the gold (Au) microelectrode applied via a mask‐assisted method. A control patch, termed e‐Patch (GR‐PCL), was prepared without drug loading, maintaining the same polymer‐to‐graphene ratio (100:2).

**FIGURE 2:**
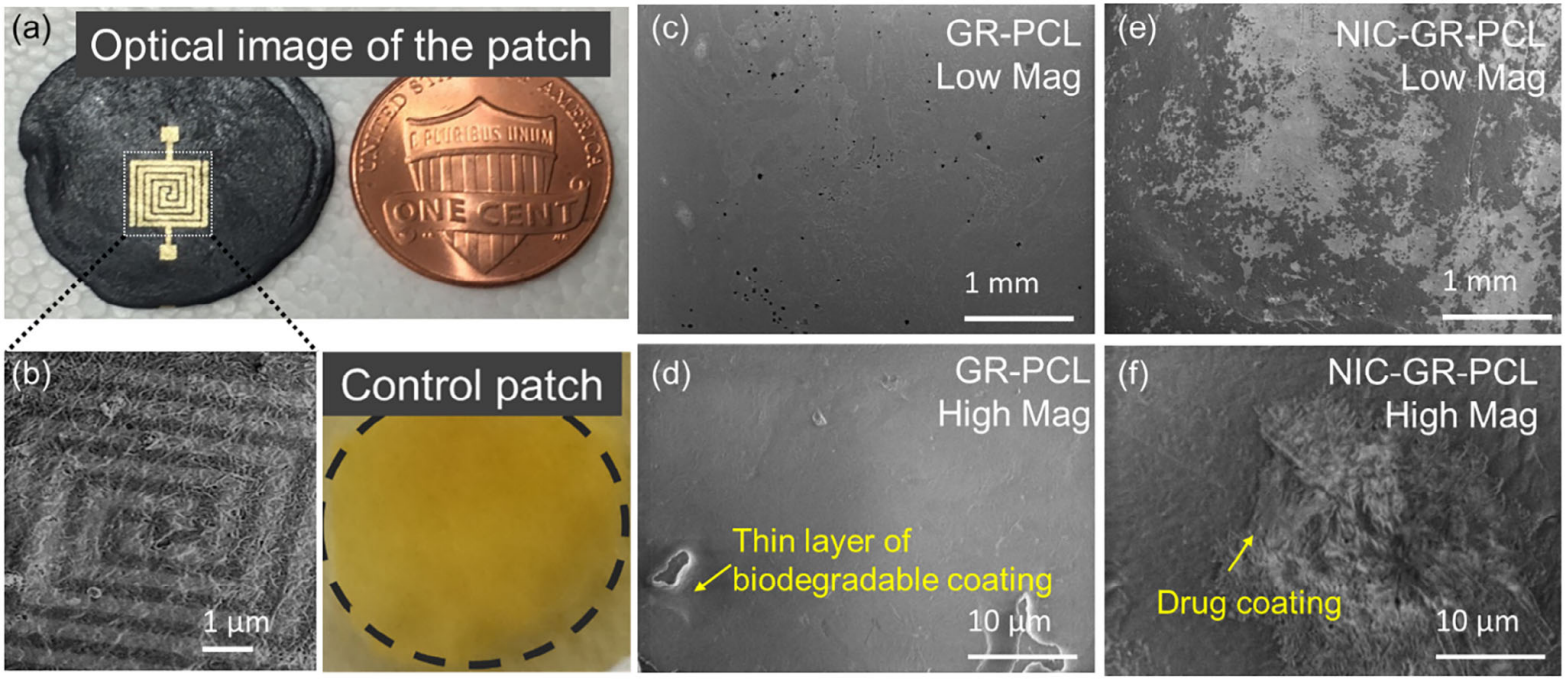
Optical and electron microscopic surface characterization of e‐Medi‐Patch and comparison with control e‐Patch. (a) Optical image of e‐Medi‐Patch compared to a one cent coin; (b) SEM features of e‐Medi‐Patch in the region of Au micro electrode decorated on drug loaded polymer‐graphene nanoplatelet composite; SEM images of GR‐PCL (c, d) without drug at low and high magnification and (e, f) with drug at low and high magnification; Distinguishable features of protruded peels of d) thin layer of polymer‐graphene nanoplatelet composite and f) drug deposited on surface.

Both e‐Medi‐Patch and e‐Patch were subsequently decorated with Au‐based microelectrodes featuring a sensing area of 1.1 × 1.1 mm^2^ within an overall patch dimension roughly equivalent to a one‐cent coin (Figure 2a). The microelectrodes were fabricated using electron‐beam evaporation under carefully controlled conditions to prevent heat‐induced damage to the patch.

Scanning electron microscopy (SEM) analysis revealed the layered structure of the prepared composites (Figure 2). Achieving highly controlled drug loading and release required understanding two key features: drug–polymer interactions and intralayer packing. The extended π‐electron system of graphene nanoplatelets promotes π–π interactions with the aromatic structure of NIC, enhancing drug retention within the matrix. Furthermore, intralayer confinement of NIC molecules enables sequential layer‐by‐layer release in response to external stimuli such as electric potential, proton concentration (H⁺), or protein‐mediated interactions.

SEM images displayed peeling polymer layers (Figure 2c,d), suggesting accessible inter‐polymer spaces where NIC molecules were distributed (Figure 2e,f). High‐magnification SEM of cross‐sectioned areas of the GR‐PCL and drug coatings in NIC‐GR‐PCL to observe similar distribution of carbon material and drug content within the layers of respective patches to conclude the homogeneity of graphene nanoplatelet distribution in PCL and distribution of drugs (Figure S1). SEM of the peeled regions in both GR‐PCL and NIC‐GR‐PCL patches showed a uniform distribution of carbonaceous material and drug content on the surface layers (Figure S1), confirming effective integration of the graphene and drug components.

**FIGURE 3:**
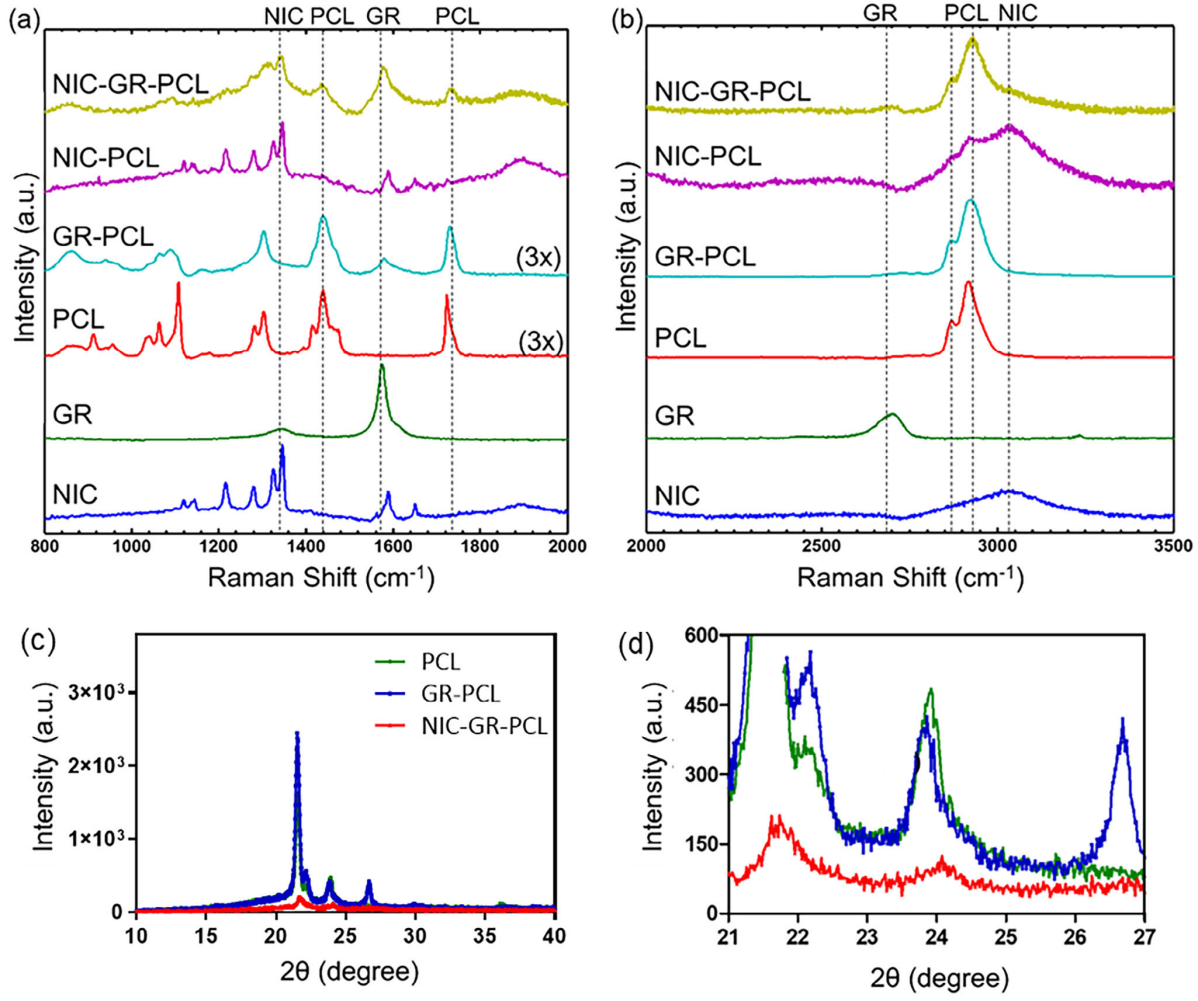
Molecular and compositional characterization of e‐Medi‐Patch and its individual components and composite with or without loaded drug. (a, b) Raman spectroscopic patterns revealing presence of chemical signatures from PCL, NIC and GR with Raman scattering representative of drug, polymer and graphene platelet, respectively; (c, d) X‐ray diffraction patterns from PCL, PCL‐GR composite without and with loaded drug to reveal variation in inter‐layer distances. d) Magnified image of the XRD region of interest.

Raman spectroscopy data revealed distinguishable chemical features of all components in prepared patches (Figure 3a, b). The Raman scattering pattern obtained from the e‐Medi‐Patch and its individual components in the range of 800–2000 (Figure 3a) and 2000–3500 cm^−1^ (Figure 3b) clearly exhibited the features of all the individual components in the e‐Medi‐Patch. The layered structure of prepared polymer‐graphene composite was proven by x‐ray diffraction (XRD) studies (Figure 3c,d) supporting the incorporation of graphene nanoplatelets and NIC in PCL. The inter‐layer spacing in e‐Medi‐Patch composite was evaluated by x‐ray diffraction (XRD) studies. From the graph it was infered that PCL (green spectrum) showed a shift in their peak positions with additional peaks due to graphene platelet (blue spectrum), which diminished significantly upon introducing NIC in the composite (red spectrum) (Figure 3c,d). This indicates that probably NIC and graphene nanoplatelets are incorporated in the inter‐layer space of PCL together due to hydrophobic interactions with aromatic rings of NIC and graphene. After the confirmation of the inter‐layer loading of NIC, drug release ability of e‐Medi‐Patch was investigated in physiological conditions of pH = 6.8 and 4.6 (Figure S2a, b), representing normal and cancer micro‐environment scenarios, respectively, and protein rich medium of fetal bovine serum (FBS), representing circulatory fluid (Figure S2c). It was found that the presence of protein content and lower pH improved the release of drug. Given the acidic microenvironment of tumors, this property may further facilitate drug release from the drug‐polymer‐graphene composite. In contrast, using these patches for topical or localized tissue applications, rather than systemic delivery, minimizes exposure to plasma proteins that could otherwise trigger premature drug release.

To achieve selective and controllable release, it was essential to integrate internal (pH‐responsive) and external (electrically driven) stimulation mechanisms.[59] With this goal, the current patch system was designed based on the principle of electric potential–mediated release of a redox‐sensitive drug, namely niclosamide (NIC). The nitroaromatic moiety in the NIC molecule renders it redox‐active and responsive to electrical stimulation property, enabling precise control over release kinetics. As a platform technology, this approach can be adapted for other therapeutics possessing redox‐responsive characteristics, broadening its potential for next‐generation smart drug delivery systems.

### 2.2 Electro‐Responsive Controlled Release of NIC From e‐Medi‐Patch

**FIGURE 4:**
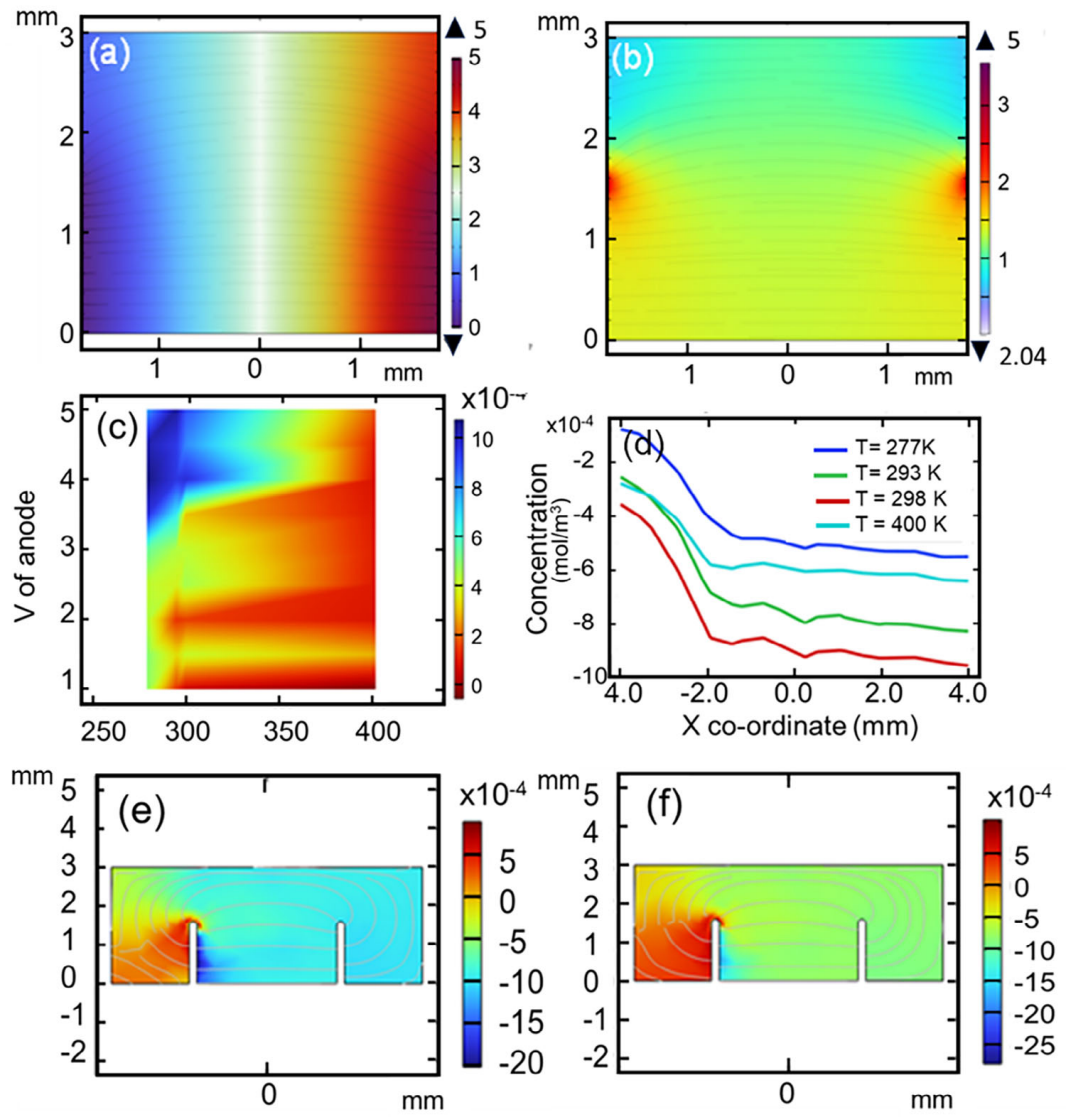
(a) The electrical potential gradient as a function of spatial location, the gradient was generated as a response to an anode voltage of 5 V. (b) The norm of the electrical field (V/m) as a function of spatial location, the gradient was generated as a response to an anode voltage of 5 V. (c) The concentration of the released drug (mol/m^3^) as a function of the spatial anode voltage and the surrounding temperature. (d) Profile of the drug release across the x‐axis (mol/m^3^) at different temperatures. The concentration of the streamline and the flux of the drug at a temperature of (e) 298 and (f) 273K.

As discussed above, the controlled drug delivery could be achieved by constant current iontophoresis which involves the application of a small electric potential (a constant current 0.1–1.0 mA/cm^2^).[60, 61, 62, 63, 64, 65, 66] The physiologically acceptable electrical currents (extra dissipated electrical energy) drive the charged permeants into the skin through electrostatic effects and make ionic drugs pass through the skin into the body with its potential gradient.[67, 68, 69, 70, 71] Figure 4a depicts the gradient of the electric potential generated due to the potential difference between the cathode and the anode. Using the patch configuration as described previously, a uniform potential gradient was generated between the anode (5 V) and the cathode. The red region near the anode had the highest electrical potential which decreased gradually towards the cathode.

Iontophoresis relies on electric field to enhance the delivery of drugs through the skin. The gradient of the electric field norm (V/m) is shown in Figure 4b. When an electric field is applied to a drug‐containing patch, it can influence the release of the drug in several ways including electroosmosis. The electric field, known as electroosmosis, can induce fluid flow within the skin and patch. As the drug molecules move in response to the electric field, they drag along the surrounding fluid, facilitating their transport across the skin. This can enhance drug release from the patch. The electric potential gradient/electric field acts as the second driving force along with the concentration gradient across the skin. The amount of drug delivered is directly proportional to the anode voltage/propagated current, the environment temperature, and the area of the skin surface in contact with the active electrode.

To get the optimum performance from our patch, we evaluated the effect of applied voltage on the drug released using computational modelling to find the optimum value. The model simulated the flow of the drug in a porous medium under the effect of the applied voltage difference between the two electrodes. We also studied the effect of temperature of the surrounding environment as another variable. Figure 4c depicts a heat map that shows the maximum concentration of the released drug as a function of both the anode voltage and temperature of the environment. The results show that the highest drug release in mol/m^2^ was found at an anode voltage of ∼5 V. Based on this finding, we used the e‐Medi‐Patch with an anode voltage of 5 V. We also evaluated the effect of environmental temperature on the release of drug to assure that the e‐Medi‐Patch would sustain extreme temperatures as well as the body temperature. This is critical for the usability of the patch as an implantable one or using it at locations with high or low temperatures. Using the optional anode voltage of 5 V, the e‐Medi‐Patch showed a good drug release at the intended range of temperatures including body temperature 37°C (310.15K), room temperature (293–298K), and temperatures higher (400K) and lower (277K) than that. The profile of the drug release across the x‐axis at an anode voltage of 5 V for the temperature of interest is shown in Figure 4d. The profile of the drug concentration across the x‐axis is temperature dependent, however, no dramatical change in the drug release was observed at various temperatures including the ones which represent environment with harsh conditions (i.e., 277 and 400K). The advantage of using an electrical potential gradient provides an improved onset time, a more rapid offset time (i.e., instantly halting drug transport once the current is switched off), and a customizable current profile (continuous and pulsative) to achieve desired drug input kinetics (Figure 4e,f).

### 2.3 In Vitro Evaluation of Functional Activity of Released NIC

**FIGURE 5:**
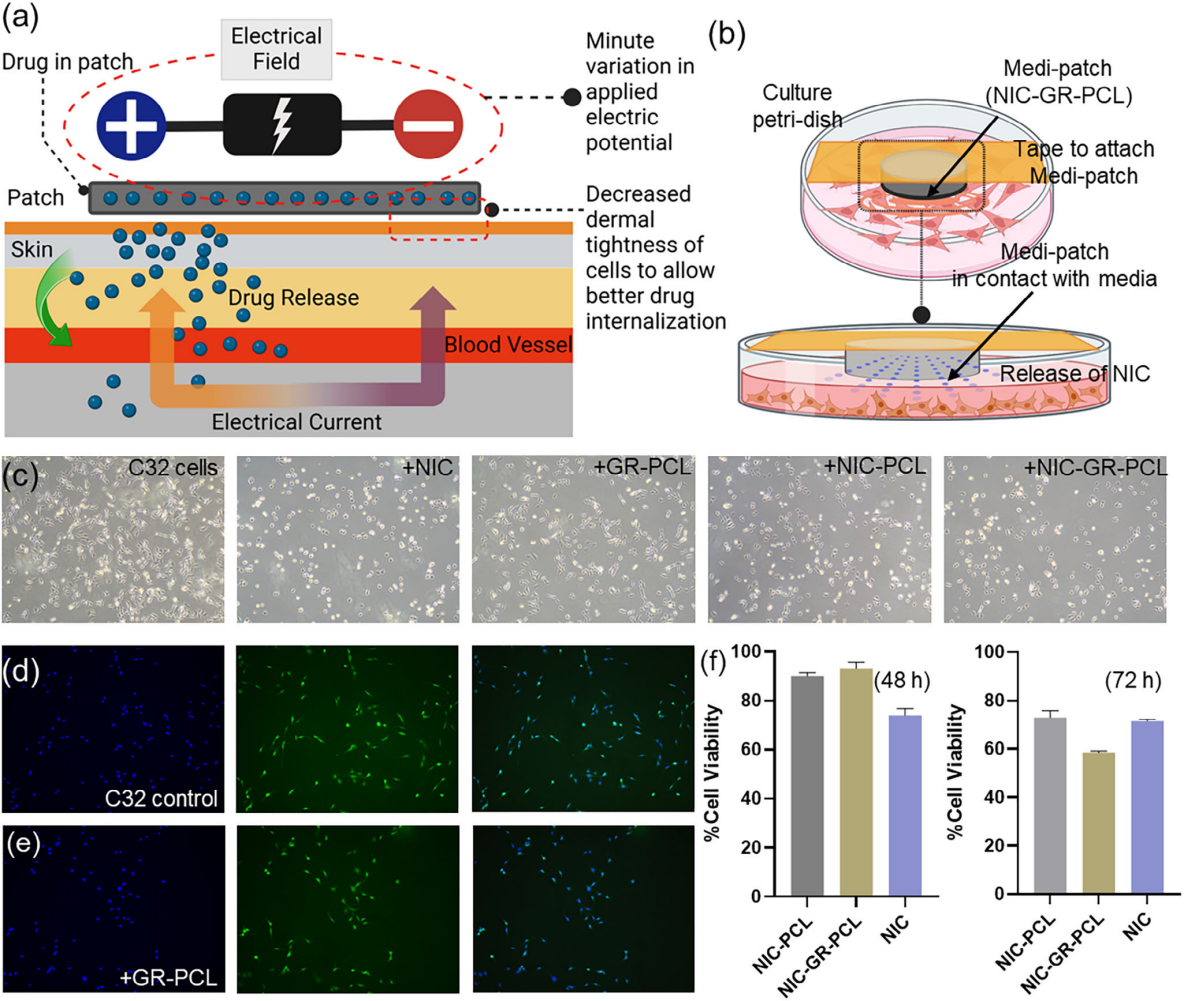
Schematic of e‐Medi‐Patch setup with plausible (a) mechanism of drug release; (b) Incubation of Medi‐patch (attached to sterilized tape) with cells for in‐vitro experiments (c) Bright field images of C32 cells after 48 h with free NIC and different formulations of medi‐patch exhibiting change in cellular morphology and growth density after 48 h treatment confirming slow and successful release of NIC over time. (d) C32 cells and (e) GR‐PCL incubated C32 cells showed negligible cell death [Hoechst33342 (blue) stains all cells, Calcein AM (green) stains live cells, Propidium iodide (red) stains dead cells] confirming insignificant effect of only GR‐PCL medi‐patch in absence of NIC (f) The % cell viability of C32 population with treatment groups post 48 and 72 h of incubation showed comparable efficiency of NIC, either released from the composites or as a free drug.

As the drug was subjected to various processes during the fabrication of e‐Medi‐Patch, an efficacy test was required to ensure the functional integrity of NIC. In vitro functional evaluation of the NIC‐loaded e‐Medi‐Patch was performed to validate the bioactivity and controlled drug release capabilities following the patch fabrication. Figure 5a depicts the hypothesized mechanism of electrically driven NIC release through the skin, showing enhanced drug penetration facilitated by the electric field. C32 melanoma cells were cultured and subsequently exposed to medi‐patches (NIC‐PCL, NIC‐GR‐PCL) and free NIC for comparative assessment to confirm functional NIC release. The e‐Medi‐Patch was attached to a sterilized tape and hanged over culture media on a cell culture petri dish (Figure 5b) so that the patch remains in contact to the media for active drug release. Brightfield microscopy images after 48 h incubation indicated clear morphological changes and a significant reduction in cell density for NIC‐treated groups compared to controls (Figure 5c). This suggests effective sustained NIC release from the patches, preserving NIC's therapeutic activity post‐fabrication. Cell viability assays using Hoechst 33342, Calcein AM and Propidium iodide staining (Figures 5d,e) confirmed negligible cytotoxic effects from GR‐PCL patches without NIC, indicating the biocompatibility of the graphene‐polymer composite. Quantitative viability assessment further corroborated these observations, demonstrating significant cell viability reduction comparable to free NIC even after 72 h of tretament (Figure 5f).

**FIGURE 6:**
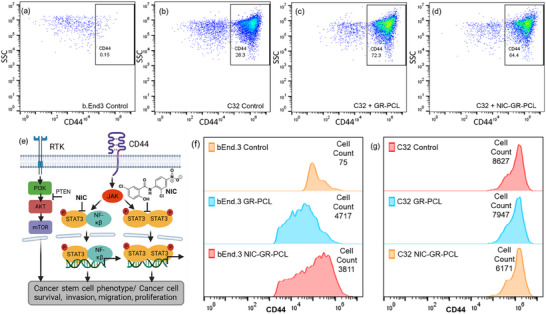
Comparative Flow Cytometry analysis of cells incubated with Medi‐patch (CD44‐PE have been used as markers) for studying (a) control non‐cancerous b.End3 cells with negligible CD44(+) cell population; (b) C32 control population (c) GR‐PCL increases CD44 expression in C32 (d) loss of stemness property (CD44) in a significant C32 population (∼11%) treated with NIC‐GR‐PCL where effect of release NIC from e‐Medi‐Patch is confirmatory (e) Mechanistic roles of NIC in inhibiting the stem cell phenotype, survival, proliferation, migration in cancer cells (f) In MFI (mean fluorescent intensities) measurements b.End3 cells showed negligible CD44 expression without treatment which increases with GR‐PCL and decreases with NIC‐GR‐PCL medi‐patch (g) C32 showed gradual decrease in CD44 expression with NIC‐medi‐patch confirming efficacy of NIC‐GR‐PCL medi‐patch.

Flow cytometric analysis was performed to assess NIC's impact on cancer stem‐like properties of cells using CD44 (PE conjugated CD44‐antibody was used) as a marker (Figure 6). Control non‐cancerous b.End3 cells showed minimal CD44 expression (Figure 6a), whereas untreated C32 cells exhibited substantial CD44‐positive populations (Figure 6b).[72] Though incubation with GR‐PCL alone did not affect cell viability, it induced increased CD44 expression levels in C32 cells (Figure 6c). Graphene exposure presumably initiates stress‐related signaling pathways that temporarily upregulate CD44, involved in cell‐cell and cell‐matrix interactions, as a compensatory or protective cellular response and may activate inflammatory signaling pathways or oxidative stress responses, contributing to increased CD44 expression to facilitate cell survival, adhesion, or migration as part of adaptive cellular processes.[73, 74] Crucially, in Figure 6d, treatment with NIC‐GR‐PCL medi‐patch markedly reduced the CD44‐positive population (∼11%), counteracting this compensatory mechanism by inhibiting crucial signaling pathways (e.g., STAT3), thereby reducing CD44 levels and cancer stem cell‐like properties. This is indicative of NIC's efficacy in diminishing cancer stemness properties. Mechanistically, NIC is known to inhibit STAT3 phosphorylation, a pathway critical for maintaining cancer stem cell phenotypes, proliferation, migration, and survival (Figure 6e). Flow cytometric mean fluorescence intensity (MFI) analyses confirmed decreased CD44 expression in C32 and b.End3 cells (in b.End3 CD44 expression increases significantly with GR‐PCL) post NIC‐GR‐PCL treatment, reinforcing NIC's role in modulating STAT3‐associated cancer pathways. Collectively, these results substantiate that the NIC‐loaded e‐Medi‐Patch retains the therapeutic efficacy of NIC, effectively reduces cancer cell viability, and inhibits explicitly cancer stem cell markers through STAT3 signaling modulation, thus presenting a promising translational tool for sustained anti‐cancer drug delivery.

### 2.4 Real‐Time Electrical Monitoring and Unidirectional Drug Release From e‐Medi‐Patch

**FIGURE 7:**
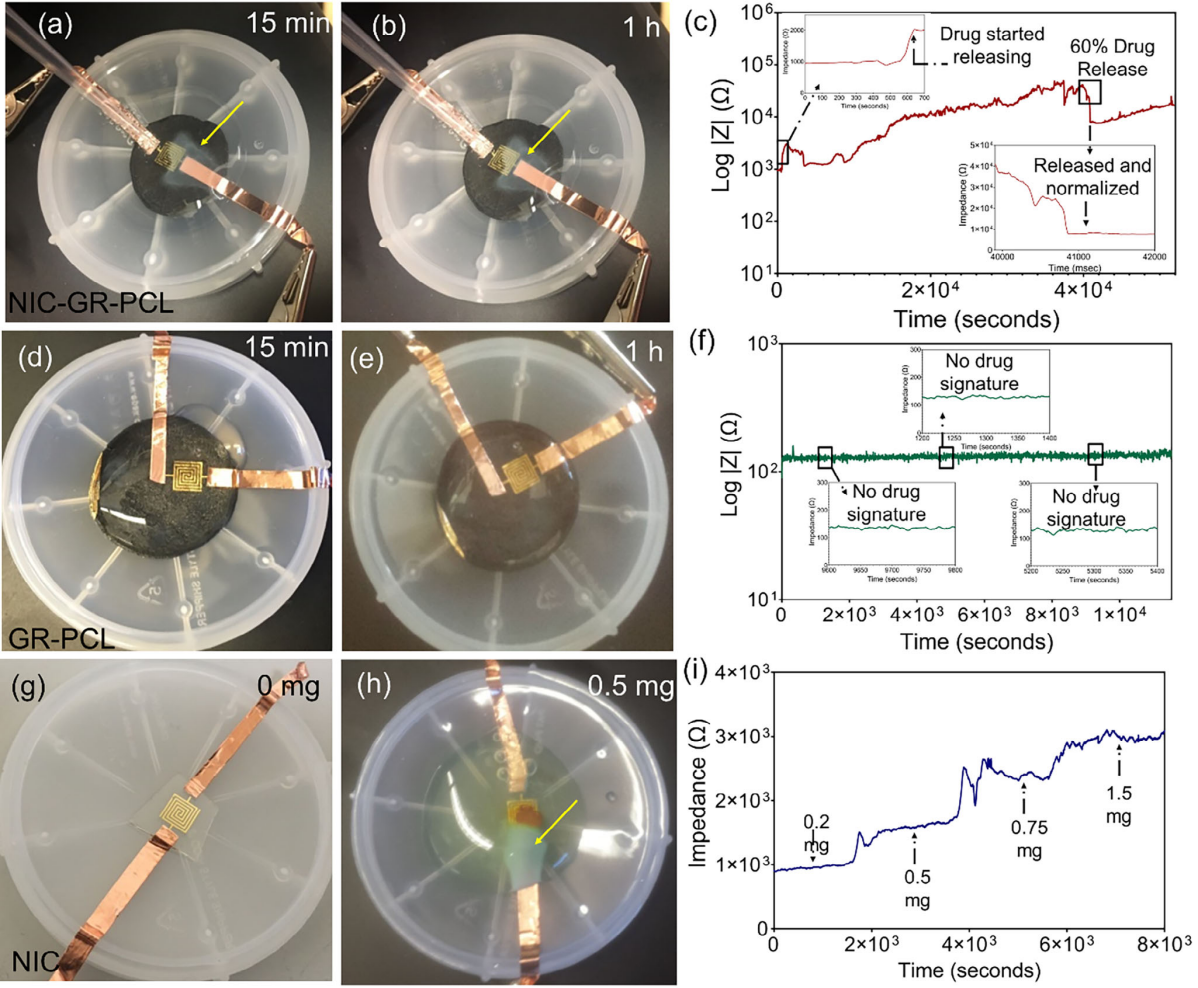
Drug release monitoring in real‐time from e‐Medi‐Patch with drug, Patch without drug and a standard sensor. Drug concentrations were verified by UV‐vis spectroscopy for NIC absorbance. (a, b) Released NIC from e‐Medi‐Patch at 15 and 60 min time points, and (c) extended monitoring till 16 h that managed to quantify a release of around 60% of loaded drug. (d, e) Patch without any drug incorporation at 15 and 60 min time points and (f) with no response to impedance monitoring with insignificant change until 3 h. (g) A micro‐electrode set‐up on glass slide and (h) after interaction with 0.5 mg of NIC. Arrow (yellow) represents the location of aggregated drug near electrodes. Experiments were performed in presence of phosphate buffer (pH 7.4). (i) Use of a control sensor for estimating the correlation between the amount of the free drug and impedance observed to create a standard plot.

The e‐Medi‐Patches were found to be highly responsive in releasing NIC in a time‐dependent manner. Our preliminary study confirmed successful tracking of the release pattern using a Bluetooth assisted impedance evaluator. Studies were performed on: (1) e‐Medi‐Patch with NIC (Figure 7a,b), (2) e‐Patch without NIC (negative control; Figure 7d,e) and (3) a standard sensor (positive control; Figure 7g,h). This efficient communication is believed to lead to convenient usage by the end user (patient) and the doctor. It will not only save time but may likely reduce multiple visits to the clinic. As this patch is planned for localized skin application, a direct release of drug at the site of application is expected to minimize undesired non‐specific interaction with neighboring healthy cells, as well.

A quantitative tracking of the released drug leads to feedback‐controlled delivery of drugs in a closed loop and real‐time monitoring that would prevent unnecessary use of bioactive agent like a chemo‐therapeutic drug. The efficient use of delivered drug would be associated with interfacing of released drug towards biological tissue which could be established by electric potential mediated unidirectional flow, which confirmed by selective direction of drug interfacing with working electrode only. Figure 7a,b shows the e‐Medi‐Patch coated with Au micro‐electrode pattern. Patch was integrated with miniaturized electronics having Bluetooth impedance analyzer. MATLAB was used to record real‐time data over a time span of 16 h. Insets of Figure 7c reveals the starting and completion period of the drug release. There was no change in resistance for the first 10–11 min, and the resistance increased significantly once the drug started releasing. Since NIC is a negatively charged species, the drug species interacted with only the sensor's working electrode (positive) as shown in the inset of Figure 7c. To study the controlled drug released from the patch, positive terminal was detached (OFF) and the drug release stopped immediately without interrupting the real‐time monitoring as shown at a time point of 4 × 10^4^ sec (Figure 7c). Drug release signatures were clearly visible throughout the live monitoring. In the second experiment e‐Patch (patch without NIC) (Figure 7d,e), real‐time experiment was demonstrated in the same fashion and by utilizing same electrode configuration. However, as expected, no drug signature was observed (Figure 7f). The recorded data was then compared with previously measured results carried out using drug loaded patch. This gave new insights on how to prepare reference patches without drugs that could be utilized in parallel to ensure the release of drugs more quantitatively. Additionally, this would help to compare the released data with the reference and protect the system from any false readings. Quantification of released drug with respect to time was estimated by fabricating another bare‐sensor (without polymer‐graphene) on glass (Figure 7g,h). This sensor was utilized to determine the resistance of the drug with respect to its known concentration. Finally, measured data (Figure 7i) was compared with e‐Medi‐Patch (Figure 7c) to estimate the unknown amount of the drug released in the process of electro‐stimulation.

During this drug release evaluation process, the calibration graph was plotted from the logarithmic scale on the y‐axis and a linear scale on the x‐axis. Impedance values were found to increase with increasing NIC concentration. A linear relationship was obtained between the known concentrations of NIC ranging from 0.2 to 1.5 mg/mL with respect to the unknown concentrations. The current limit of detection was determined to be 0.1 mg/mL of NIC based on the known concentration and indicates the possibility to monitor the drug release of even less than 1 mg. It is clear from these results that drug monitoring could be possible in singular manner and under competitive environment with e‐Medi‐*Patch*. Released drug concentration could be verified by UV‐Vis absorption determination at λ_max_ of 375 nm.

### 2.5 In Vivo Tumor Growth Regression Efficiency of e‐Medi‐Patch

**FIGURE 8:**
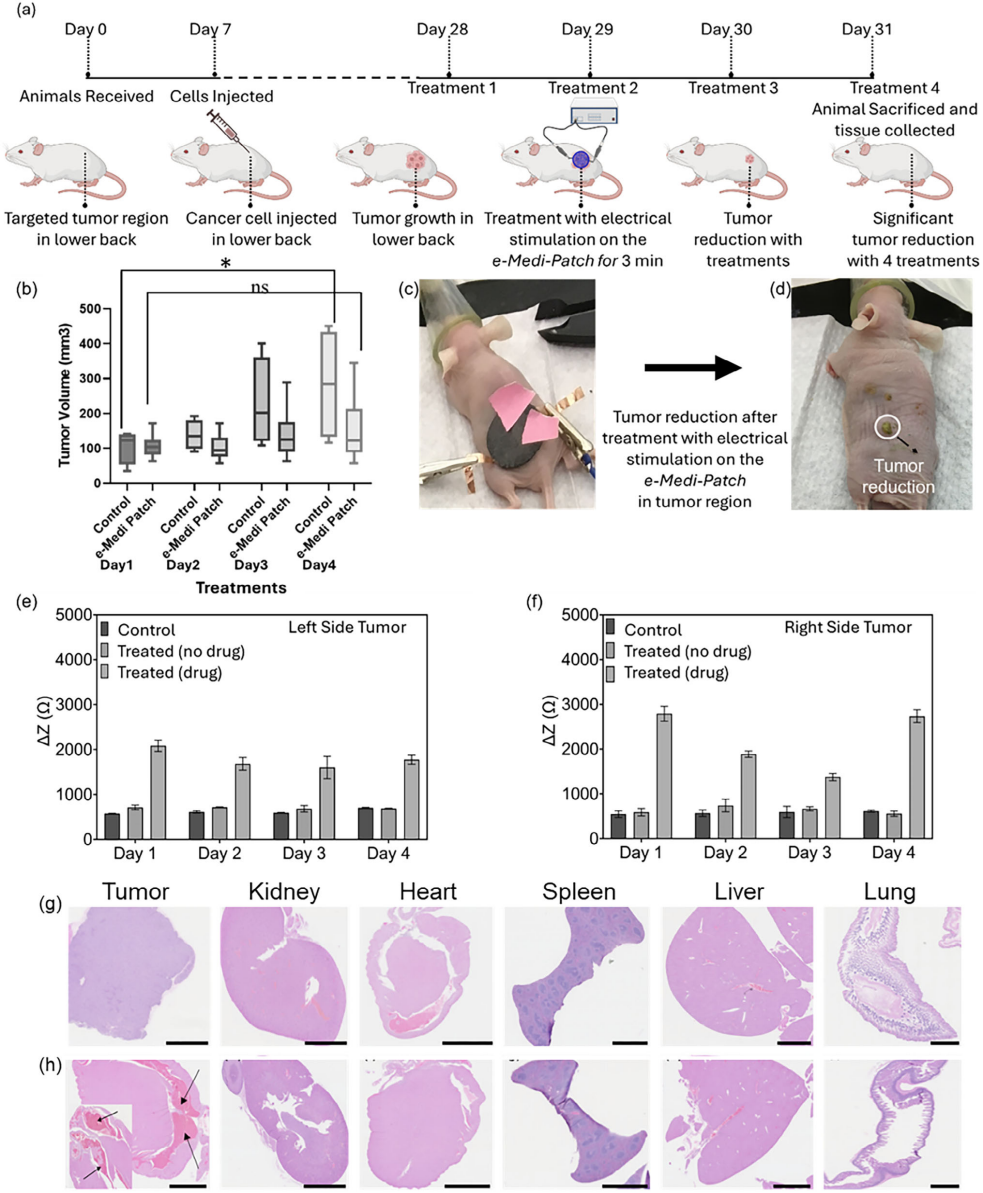
In vivo use of e‐Medi‐Patch to evaluate the efficiency in xenograft melanoma nude mouse model. (a) Timeline of experimental procedure with schematic of ‐mouse model. (b) Variation in tumor volume from the group of animals without and with treatment of e‐Medi‐Patch. A representative animal (c) during and (d) after, application of e‐Medi Patch treatment. Change in the impedance values (ΔZ) of the e‐Medi‐Patch after 3 min of treatment on the (e) left side tumor (f) right side tumor. Control indicates animals (N = 2) not treated with e‐Medi‐Patch. Treated (no drug) indicate animals (N = 2) treated with e‐Patch without drug (niclosamide). Treated (drug) indicates animals (N = 3) treated with e‐Medi‐Patch with drug (niclosamide)‐. p value of < 0.05 has been represented as * and > 0.05 as ns. (g) Representative H&E‐stained sections of tissues collected from control animals and (h) e‐Medi‐Patch treated groups. Tissues were collected from C32 cell xenograft tumor, kidney, heart, spleen, liver and lungs. Arrow heads represent loss of tissue integrity in e‐Medi‐Patch treated niclosamide. (Scale: 2 µm).

Efficiency of e‐Medi‐Patch in animal model was performed using a xenograft nude mice model. The e‐Medi‐Patch with drug (treated) and control e‐Patch without drug (non‐treated), were subjected to electrical pulse on the grown tumors on 28, 29, 30 and 31st day after injecting the cells (Figure 8a). Tumor volumes were recorded till the end of the study on 31st day after first injection of the cells (Figure 8b–d). Treatment was executed for 3 min on each day of application with electrical potential, for 4 consecutive days (Figure S3). It was observed that the e‐Medi‐Patch with drug had a significant change in the impedance after 3 min of treatment in comparison to control e‐Patch without the drug in both left and right side tumors (Figure 8e,f). This could be due to the amount of drug released from the patch. The animals were sacrificed at the end of the study and tumor tissues along with tissues from other major organs were collected and fixed for H&E staining. A significant change (p < 0.05) in tumor size for e‐Medi‐Patch treated animals was reported at the end of the experiment (Figure 8b). However, there was insignificant change in body weights for both the treated and non‐treated groups (Figure S4).

### 2.6 Gross Pathology and H&E Analysis

On day 31, post‐treatment completion, all animals were sacrificed, and tumors along with major organs including liver, kidney, heart, spleen, and lungs were harvested for histo‐pathological evaluation (Figure 8g,h). Histological analysis revealed pronounced eosinophilic staining in tumor sections from the e‐Medi‐Patch treated group (Figure 8h), indicative of enhanced cellular damage and necrosis, compared to the relatively intact architecture in tumors from animals treated with the drug‐free e‐Patch (Figure 8g). This aligns with the observed reduction in tumor volume exclusive to the e‐Medi‐Patch cohort, supporting its therapeutic efficacy. Importantly, histological examination of all major organs showed preserved tissue architecture without signs of significant inflammation or necrosis across both treatment groups. These findings underscore the systemic biocompatibility and minimal off‐target toxicity of the patch formulations, validating the safety profile of the e‐Medi‐Patch platform for localized drug delivery.

## 3 Conclusions

This study introduces an electro‐responsive graphene‐polymer nanocomposite patch as a multifunctional platform for externally regulated and quantitatively monitored drug release. By coupling hierarchical graphene‐polymer architectures with Au microelectrodes and Bluetooth‐enabled impedance sensing, the system achieves unprecedented control over dosage, directionality, and feedback monitoring of redox‐active drugs. The scalable melt‐blending fabrication and robust electro‐stimulatory release mechanism position this platform as a versatile material solution for wearable therapeutics, adaptable to diverse biomedical applications including cancer, wound healing, and infectious diseases. More broadly, this work highlights the convergence of nanomaterials and bioelectronics in creating intelligent, feedback‐controlled therapeutic platforms. We are now investigating PCL blends with elastomeric and breathable polymers such as polyurethane, PEG‐PLA copolymers, and medical‐grade silicones to improve mechanical adaptability and user comfort while retaining the electroactive performance of the system. Here, a proof‐of‐concept polymer‐graphene composite was engineered to encapsulate the redox‐active anticancer drug niclosamide, with potential adaptability for loading other redox‐responsive therapeutics. In melanoma xenograft nude mice, only a few sessions of mild electrical stimulation via the patch led to a marked reduction in tumor volume, demonstrating the efficacy of this electro‐responsive delivery strategy.

## 4 Materials and Methods

Poly(caprolactone) (50 000 MW) was purchased from Polysciences, Inc. Graphene nanoplatelets were purchased from Strem Chemicals (Newburyport, MA, USA). Niclosamide was procured from AKA Scientific Inc. (Union City, CA, USA). Human melanoma cell line C32 was obtained from American Type Culture Collection (ATCC). EMEM (Sigma) medium with 10% fetal bovine serum (FBS) (Seradigm, US)) and 1% Penicillin+Streptomycin (PenStrep) (Lonza) was used for cell culture. Anti‐CD44 (FITC) (FITC‐conjugated CD44 antibody) was procured from Thermo Scientific. Trypsin (EDTA 0.02%, dextrose 0.05%, and trypsin 0.1%) was used for passaging the cells after achieving ∼80% of confluence. Tetrazolium salt 3‐[4,5‐dimethylthiazol‐2‐yl]‐2,5‐diphenyltetrazolium bromide was obtained from Sigma. Trypsin and Tetrazolium salt were purchased from Sigma‐Aldrich. All the organic solvents were used without further purification. Flow assisted cell sorting was performed on an iCyt Reflection machine from iCyt Mission Technology equipped with software Win List 3D.

### 4.1 Preparation of GR‐PCL Composite

Graphene nanoplatelets of 5–10 nm thickness and 25 µm of particle size were used for making the composite with PCL which was purchased from Strem Chemicals (Newburyport, MA, USA). It was found to be practically insoluble in aqueous medium and could easily mix in melted form of PCL at 65°C. To further ensure the mixing of graphene nanoplatelets in PCL, during process of melt‐mixing water of ∼65°C was added to ensure all of the highly hydrophobic graphene platelets preferring to be in melted PCL rather than hot aqueous medium.

### 4.2 Fabrication of Electro‐Responsive Medicinal Patch (e‐Medi Patch)

An optimized weight ratio of 100:2:1 (250 mg of PLAPCL and 5 mg graphene nanoplatelet (5 mg) and, loaded with 2.5 mg of NIC), per patch, was used for a melt blending method to prepare e‐Medi Patch. A control patch, called as e‐Patch, was prepared without loading of the drug but having same optimized weight ratio of polymer and graphene nanoplatelet of 100:2. Au based micro‐electrode with sensing area of 1.1 ´x 1.1 mm^2^ was coated by using an electron‐beam evaporator directly on the patches.

### 4.3 Scanning Electron Microscopic Studies

The scanning electron microscopic (SEM) investigation could be used to reveal the layered structures of prepared patches. SEM was performed on GR‐PCLe‐ Patch and NIC‐GR‐PCLe‐Medi Patch samples without printed Au electrode and NIC‐GR‐PCLe‐Medi Patch coated with Au electrode, with a JEOL 6060 Scanning Electron Microscope with an acceleration voltage of 5 kV.

### 4.4 Raman Scattering Studies

Raman spectroscopic studies were performed on various patches and its individual components. Raman spectra were recorded in the range of 800–3500 cm^−1^ by using a 532 nm laser for 60 s at 0.2% laser power. For each sample multiple spectra were recorded and averaged.

### 4.5 X‐Ray Diffraction Studies

The inter‐layer spacing in e‐Medi‐Patch and e‐Patch was evaluated by X‐ray diffraction studies. XRD of individual samples was performed using the reflection method with a Siemens‐Bruker D5000 diffractometer. The X‐ray beam was generated with a Cu anode and the Cu‐Kα beam of wavelength 1.5418 Å was used for the experiments. Scans were performed for 2Ɵ range of 10–40.

### 4.6 Passive Drug Release Studies

The passive drug release ability of e‐Medi‐Patch was investigated in physiological conditions of pH = 6.8, 4.6 representing normal and cancer micro‐environment scenarios, and protein rich medium of fetal bovine serum, representing circulatory fluid. UV‐Vis spectra were recorded on Genesys 10S UV‐Vis Spectrophotometer machine to determine the absorbance values at different time points. Generated standard plots were used for estimating unknown values of the released drug, passively in different mediums.

### 4.7 Electro‐Responsive Release of NIC From e‐Medi Patch in Silico

In vitro evaluation for functional activity of released NIC from e‐Medi Patch was studied. A 2D culture of 40,000 human melanoma cells (C32) was cultured in a 24 well plate using 400 µL EMEM medium with 10% FBS per well for 24 h before being incubated with pieces of NIC loaded in PCL and PCL‐graphene composites of 1 mg/well (∼2.5 mg/mL of medium). For control experiments, non‐cancerous brain epidermal b.End3 cells were cultured in DMEM medium with 10% FBS and 1% Pen‐Strep. Cells were incubated for 72hdifferent time points before visualizing the cell growth density and morphological changes. At the end of the incubation period a cell viability test was performed by using MTT assay. Free NIC of approximately same concentration (∼37.5 µM) was used as positive control whereas non‐treated cells were used as negative controls.

To investigate the mechanistic impact of NIC released from the e‐Medi‐Patch, a flow cytometry (FACS) analysis was conducted using confluent T25 flasks of C32 melanoma and b.End3 endothelial cells. Each cell population was enriched for CD44‐positive cells prior to treatment. For experimental groups, CD44(+) enriched C32 cells were incubated with e‐Medi‐Patch (5 mg/well, equivalent to ∼2.5 mg/mL in medium) for 24 h. Parallel untreated CD44(+) enriched C32 samples served as controls and were incubated under identical conditions without the patch. Post‐incubation, cells were trypsinized, washed with PBS, and stained with anti‐CD44‐PE antibody (1 µg/mL) for 30 min at 4°C in the dark. To assess cell viability and exclude dead cells, 7‐AAD was added immediately prior to acquisition. Data were acquired on a flow cytometer, and 7‐AAD‐negative (live) populations were gated for subsequent analysis. Histograms of CD44‐PE fluorescence intensity were plotted to quantify changes in CD44 expression between treated and untreated groups. This approach enabled simultaneous assessment of both phenotypic changes in CD44 levels and cell viability, confirming NIC‐mediated modulation of cancer stem‐like markers.

### 4.8 Real‐Time Electrical Monitoring and Unidirectional Release of Drug From *e*‐Medi Patch

A Bluetooth assisted impedance evaluator was used for this study. The electrodes from the patch were interfaced with Bluetooth based impedance analyzer module and drug release was monitored. The impedance analyzer was assembled using a microcontroller (ATmega328P) to control the input voltage of 5 V, Bluetooth module (HC‐05), and LCD to display the output resistance across the biosensor chip. MATLAB was utilized to run the Bluetooth data and real‐time monitoring. The Bluetooth device used in this study was a commonly used Bluetooth module that has a standard baud rate of 9600 and operates at 2.4 GHz for its transmission with an input voltage of 3.3 V. One electronic module was utilized to send electronic signals to the actuating electrodes for drug release.

### 4.9 In Vivo Tumor Study

The studies were conducted with two groups of five xenograft nude mice animals, each injected with 5 × 10^6^ C32 cells subcutaneously on lower back and followed for three weeks till tumors reached to a considerable size of ∼100 mm^3^). Experiments were performed using approved protocol # 14045 at the University of Illinois at Urbana‐Champaign. The e‐Medi‐Patch with drug and control e‐Patch without drug, were subjected to electrical pulse, on the grown tumors on 28, 29, 30 and 31st day after injecting the cells. A vernier caliper was used to monitor the change in tumor volume. Electrical potential was applied across the patch with optimal conditions without harming the animal tissues for 3 min on each day of the treatment, for 4 days. Afte the completion of the study on 31st day, the tumor tissues along with tissue from other major organs were collected after sacrificing the animal and fixed before using for H&E staining.

### 4.10 In Vivo Tumor Growth Regression Efficiency of *e‐Medi Patch*

Efficiency of *e‐Medi‐Patch* in animal model was performed using a xenograft nude mice model. Two groups of five nude mice animals each were injected with 5 × 10^6^ C32 cells subcutaneously on lower back and followed for three weeks till tumor reaches to a considerable size of ∼100 mm^3^). Experiments were performed using approved protocol # 14045 at the University of Illinois at Urbana‐Champaign. The e‐Medi‐patch and control e‐patch, i.e., patches with drug treated with electrical pulse and no treatment, respectively, were applied on the grown tumors on 28, 29, 30 and 31st day after injecting the cells. Tumors were followed for varying volume till 31^st^ day after first injection of the cells. Treatment was executed for three min on each day of application. Electrical potential was applied with optimal conditions without harming the animal tissues.

### 4.11 Statistical Analysis

Graphing and statistical analysis were performed using GraphPad Prism. One‐way ANOVA was used for statistical analysis using data from day one as control. A p value of < 0.05 has been represented as * whereas > 0.05 was considered ns.

## Conflicts of Interest

The authors declare no conflict of interest.

## Author Contributions

D.P. conceived the idea and D.P., S.K.M., K.D., and P.S. designed and performed the experiments. D.P., S.K.M., K.D., and P.S. analyzed the data and wrote the manuscript. T.A., M.S.K., M.A., and P.M. helped with manuscript preparation and experiments.S.K.M, K.D., P.S. contributed equally for the manuscript. TA, M.S.K. contributed equally. All authors approved the manuscript.

## Supporting information




**Supporting File**: adhm70702‐sup‐0001‐SuppMat.docx.

## Data Availability

The data that support the findings of this study are available in the supplementary material of this article.
