# Supplementary material for: Electro‐Stimulated Graphene‐Polymer Nanocomposites Enable Wearable Patches With Feedback‐Controlled Drug Release
Source: Adv Healthc Mater. 2025 Dec 29;15(15):e05894. doi: 10.1002/adhm.202505894 (PMC13088759; doi:10.1002/adhm.202505894)
Supplement: Supplementary file 1 — Supporting File: adhm70702‐sup‐0001‐SuppMat.docx. [file ADHM-15-0-s001.docx]

Supporting Information

**Electro-stimulated Graphene-Polymer Nanocomposites Enable Wearable Patches with Feedback-Controlled Drug Release**

Santosh K. Misra,^a,†^ Ketan Dighe,^b,†^ Pranay Saha,^c,†^ Teresa Aditya,^c,‡^ Muhammad S. Khan,^d,‡^ Maha Alafeef,^c,e^ Parikshit Moitra,^c^ and Dipanjan Pan^b,c,f,g,h,^*

^a^ Bioengineering Department, University of Illinois at Urbana-Champaign, Urbana, 61801, USA

^b^ Department of Biomedical Engineering, The Pennsylvania State University, University Park, PA 16802, USA

^c^ Department of Nuclear Engineering, The Pennsylvania State University, Hallowell Building, University Park, Pennsylvania 16802, USA

^d^ Cardiovascular Research and Training Institute, The University of Utah, Salt Lake City, UT 84112, USA

^e^ Biomedical Engineering Department, Jordan University of Science and Technology, Irbid 22110, Jordan

^f^ Department of Materials Science and Engineering, The Pennsylvania State University, University Park, PA 16802, USA

^g^ Department of Chemistry, The Pennsylvania State University, University Park, PA 16802, USA

^h^ Huck Institutes of Life Sciences, Millennium Science Complex, The Pennsylvania State University, University Park, PA 16802, USA

^†^,^‡^ Equal contribution


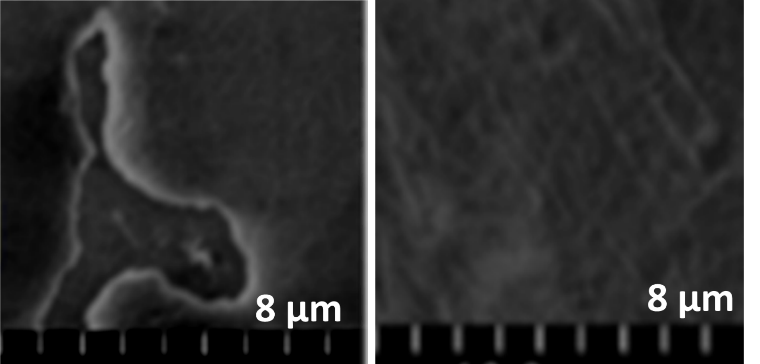

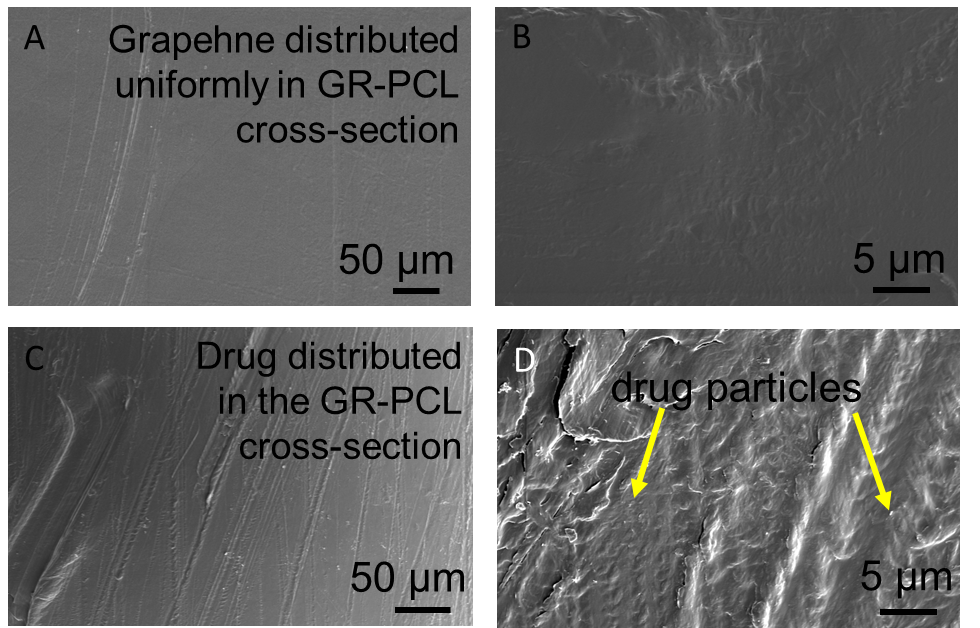


E

**Figure S1.** High magnification SEM images from the cross-sectioned areas of the (A-B) GR-PCL and (C-D) drug coatings in NIC-GR-PCL show uniform distribution of carbon material and drug content in the layers of the respective patches. (E)High magnification SEM images from the pealed areas of the GR-PCL (left) and drug coatings in NIC-GR-PCL (right) to observe similar distribution of carbon material and drug content on the top layer of respective patches.


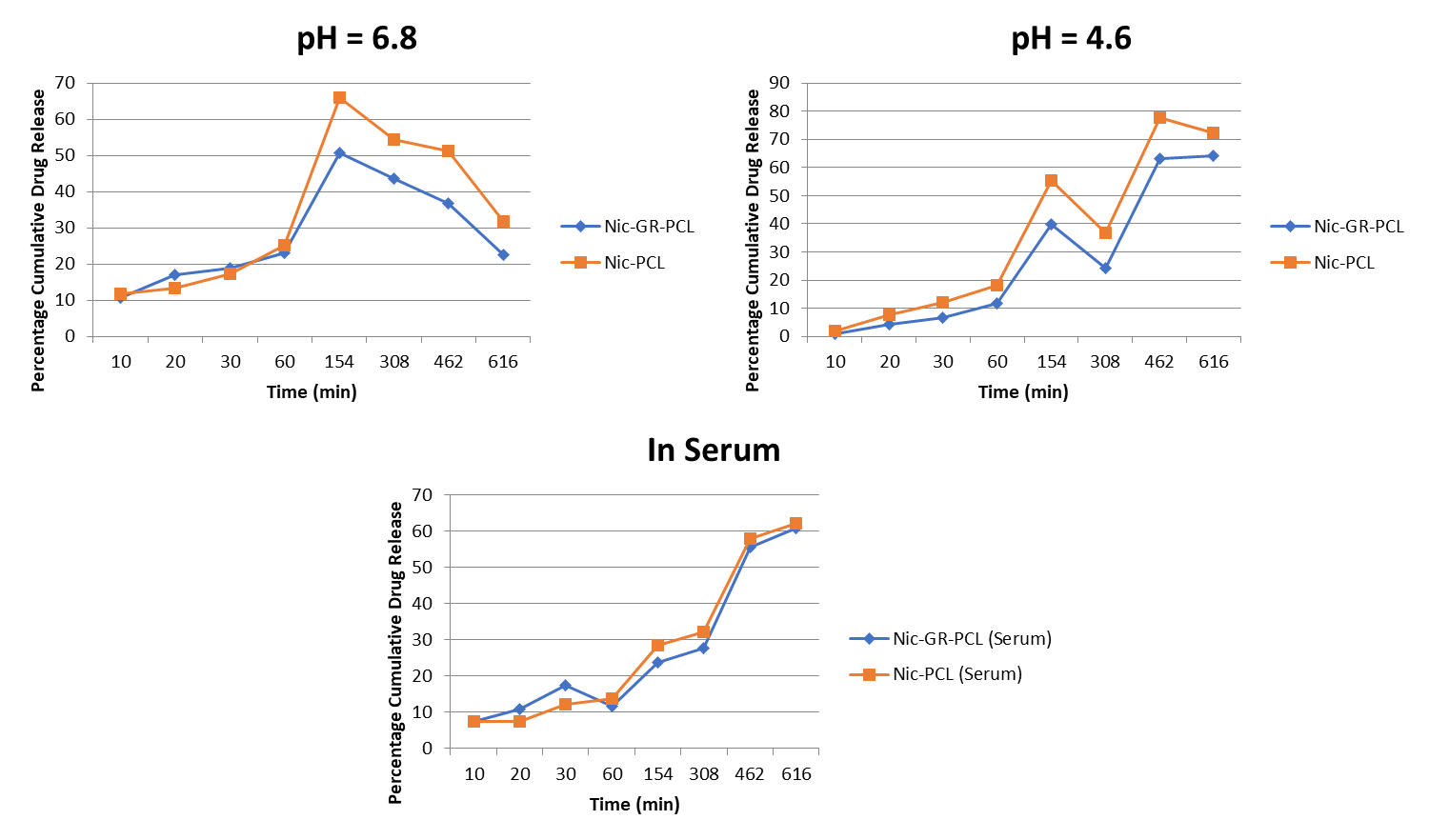


a

b

c

**Figure S2.** Release pattern of NIC from patch having polymer (Red spectra) and polymer-graphene nanoplatelet composite (Blue spectra) in different simulated physiological conditions of (a) pH = 6.8; (b) pH = 4.6 and (c) serum with proteins.


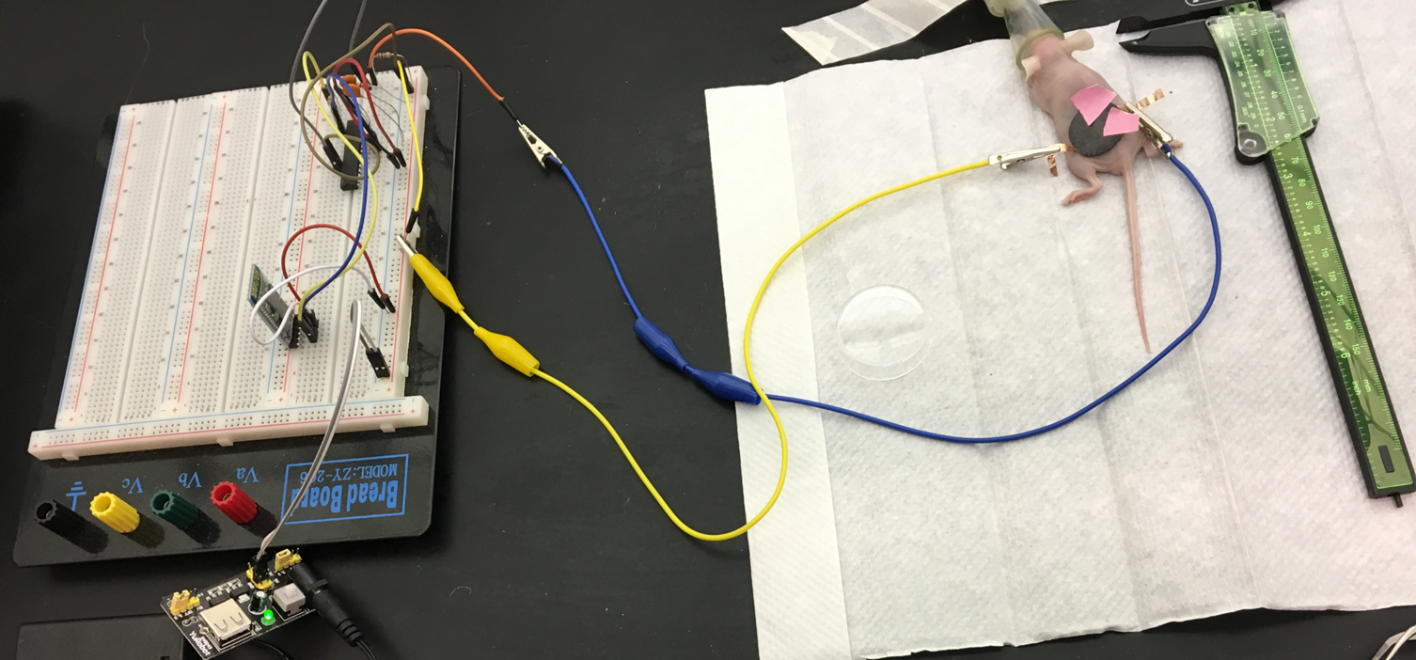

**Figure S3.** Experimental setup showing e-Medi Patch treatment being performed on the animal.

**Figure S4.** Average weight of animals recruited for the study. No significant difference was reported in body weight of animals during application of e-Medi Patch. Control group of animals also did not show any significant change in body weight during this time period though tumor size grew to a significant level.
